# Transcriptomic Analysis of *Chilo suppressalis* (Walker) (Lepidoptera: Pyralidae) Reveals Cold Tolerance Mechanisms Under Parasitism Stress

**DOI:** 10.3390/insects16090907

**Published:** 2025-09-01

**Authors:** Chuan-Lei Dong, Elyar Abil, Rong Ji, Yu-Zhou Du, Ming-Xing Lu

**Affiliations:** 1 International Research Center for the Collaborative Containment of Cross-Border Pests in Central Asia, Xinjiang Key Laboratory of Special Species Conservation and Regulatory Biology, College of Life Sciences, Xinjiang Normal University, Urumqi 830054, China; dongcl@xjnu.edu.cn (C.-L.D.);; 2 Tacheng, Research Field (Migratory Biology), Observation and Research Station of Xinjiang, Tacheng 834700, China; 3 College of Plant Protection & Institute of Applied Entomology, Yangzhou University, Yangzhou 225009, China; 4 Jiangsu Province Engineering Research Center of Green Pesticides, Yangzhou University, Yangzhou 225009, China

**Keywords:** *Chilo suppressalis*, cold tolerance, transcriptome, heat shock protein, endoplasmic reticulum

## Abstract

Parasitoids exhibit remarkable abilities to manipulate host physiology, ensuring offspring survival and development. This study investigated the molecular mechanisms underlying how the parasitoid *Cotesia chilonis* modulates cold tolerance in its host, the rice stem borer *Chilo suppressalis*, using transcriptome sequencing. We found that the host larvae’s supercooling point was lowest at 3 days post-parasitism but increased significantly by day 4. Transcriptome analysis identified 507 differentially expressed genes (DEGs), including 235 up-regulated by parasitism. Functional enrichment revealed that these DEGs were primarily associated with ribosome biogenesis, protein processing in the endoplasmic reticulum (ER), and oxidative phosphorylation under parasitism stress. Notably, 24 DEGs linked to temperature tolerance were predominantly heat shock proteins (HSPs) and calcium signaling-related genes. The reliability of transcriptome data was confirmed via RT-qPCR for eight randomly selected DEGs. Functional assays demonstrated that parasitism stress significantly inhibited ER activity. However, HSP expression did not significantly affect ER activity or cytosolic Ca^2+^ concentration in the hemolymph cells of *C. suppressalis* larvae. This research provides insights into the complex physiological and molecular mechanisms through which *C. suppressalis* responds to parasitism stress, particularly concerning cold tolerance modulation.

## Simple Summary

Parasitoid wasps like *Cotesia chilonis* manipulate their host, the rice stem borer *Chilo suppressalis*, to ensure their offspring’s survival. In this study, we used transcriptome sequencing to explore how *C. chilonis* alters the cold tolerance of *C. suppressalis* larvae. We found that the host’s cold tolerance peaked 3 days (SCP values in −6.93 °C) after parasitism but dropped sharply by day 4 (SCP values in −4.25 °C). Analysis revealed 507 genes with altered expression, including 235 induced by parasitism. These genes were mainly involved in ribosome function, protein processing in the endoplasmic reticulum (ER), and energy production. Notably, genes related to temperature tolerance, such as heat shock proteins (HSPs) and calcium signaling, were also affected. Further experiments confirmed that parasitism stress reduced ER activity but did not significantly impact HSP expression or calcium levels in the host. This study deepens our understanding of the complex molecular and physiological changes in *C. suppressalis* when parasitized.

## 1. Introduction

Parasitoids manipulate the host behavior or physiological processes to benefit the development and survival of their offspring [1]. It has been shown that parasitoids activate insulin and insulin-like peptide (ILP) signaling pathways to reduce host energy expenditure and free water content, thereby increasing host cold tolerance [2,3]. Xing et al. (2023) found that *Spodoptera frugiperda* feeding behavior was modulated by *Microplitis manilae* to reduce uptake of heterogeneous ice nuclei and to promote the synthesis of small-molecule cryoprotectants to improve host cold tolerance [4]. In addition, parasitism stress stimulates the expression of specific genes, especially heat shock proteins (HSPs), which increase the temperature tolerance of the host. Understanding the impact of parasitism stress by measuring the supercooling point (SCP) of individuals has proven to be useful in predicting long-term temperature-tolerant behavior of insects [5]. For instance, when *Sarcophaga crassipalpis* was parasitized by *Nasonia vitripennis*, the up-regulation of *ScHsp23* and *ScHsp26* expression levels was accompanied by a significant decrease in the SCP, and the host showed strong cold tolerance [6].

The host hemolymph plays an essential role in the growth and development of most endoparasitoids [7]. Studies indicate that endoparasitoids exert parasitism stress on hosts by injecting secreted effector factors (such as venom, polydnavirus, teratocyte, etc.), which disrupt the host’s development, immune system, and metabolism, ultimately leading to changes in the host’s supercooling point [8]. This process also involves the rearrangement of cellular structure and polarized redistribution of organelles [9]. For example, parasitism stress induces irreversible mitochondrial outer membrane permeabilization (MOMP), leading to increased intracellular Ca^2+^ concentrations and endoplasmic reticulum (ER) stress [10]. Sustained ER stress leads to disruptions in protein processing and glycogen and lipid production, which directly affect host cold tolerance. However, the effects of parasitism stress on ER activity and intracellular Ca^2+^ concentrations in host hemolymph cells, and their relationship with host cold tolerance, have been rarely reported.

The rice striped stem borer, *Chilo suppressalis* (Walker) (Lepidoptera: Crambidae), is a widespread rice pest in China, and its population size is closely related to environmental factors and natural enemies [11,12]. *Cotesia chilonis*, an obligate endoparasitoid, parasitizes up to 90% of overwintering *C. suppressalis* larvae in the field [13]. However, it is unclear if *C. chilonis* enhances the host’s cold tolerance and aids the parasitoid’s overwintering, as well as the underlying molecular mechanisms. In this study, we initially quantified the changes in the SCP of *C. suppressalis* larvae at various time intervals following parasitism by *C. chilonis*. Subsequently, transcriptome sequencing (RNA-seq) was used to identify genes associated with the temperature tolerance of *C. suppressalis*, and the molecular functions of these genes were further validated. This study enhances understanding of the molecular mechanisms in host–parasitoid interactions and parasitism-induced changes in host temperature tolerance, offering a theoretical foundation for the improved biological control of *C. suppressalis*.

## 2. Materials and Methods

### 2.1. Insects

The *C. suppressalis* larvae and its obligate endoparasitoid *C. chilonis* were collected from fields in Yangzhou (32.39° N, 119.42° E). *Chilo suppressalis* larvae were reared on an artificial diet and maintained at 27 ± 1 °C, 75 ± 5% RH, and 16 h light/8 h dark in the laboratory. *Cotesia chilonis* adults were fed with a 10% (*v*/*v*) honey–water solution and propagated using fifth instar *C. suppressalis* larvae. More than three generations of *C. suppressalis* and *C. chilonis* were continuously reared prior to experiments.

### 2.2. Determination of Supercooling Point (SCP)

One fifth instar *C. suppressalis* larva from the same batch was placed in a test tube with two female and one male *C. chilonis* adults to facilitate parasitism at 27 °C in darkness. On completion of parasitism, *C. suppressalis* larvae were reared normally, and the supercooling point (SCP) was measured every 24 h for nine consecutive days using a thermocouple connected to an automatic temperature recorder (UT-325, Uni-Trend Technology, Shenzhen, China). The thermocouple tip in contact with the larva was secured in an Eppendorf with cotton, and the larva was then placed at −20 °C. After the larva temperature dropped below 0 °C, their body fluids froze due to the release of heat, and the temperature rose abruptly, with the lowest temperature being recorded as the SCP [14]. The unparasitized *C. suppressalis* larvae from the same batch were used as controls. All treatments included 20 individuals.

### 2.3. RNA Isolation and Transcriptome Sequencing

*Chilo suppressalis* larvae were collected separately after 3 and 4 days of parasitism, frozen with dry ice, and sent to Beijing Biomarker Technology Co., Ltd. (Beijing, China). Samples were only collected for subsequent RNA extraction and sequencing after we directly witnessed a successful parasitism event by the female *Cotesia chilonis*. Briefly, high-purity and high-integrity RNA of all samples was examined prior to cDNA library construction and sequencing. Sequencing libraries were prepared using the Illumina TruSeq RNA Sample Preparation Kit (Illumina, San Diego, CA, USA), and samples (*n* = 9) were sequenced using an Illumina HiSeq™ 2500 instrument as recommended by the manufacturer.

### 2.4. Transcriptome Assembly and Gene Expression Analysis

Raw transcriptomic data were preprocessed to remove clip adapter sequences and filter low-quality reads. To obtain a full-length unigene library, the high-quality clean reads were de novo-assembled using Trinity software (Version 2.1.1). Then, unigene annotation was performed using the Nr, Nt, Swiss-prot, COG, Pfam, and KEGG databases, considering an E-value cut off of 10^−5^. Unigenes were automatically assigned an internal ID.

The fragments per kilobase of transcript per million mapped fragments (FPKM) was calculated to find the area of candidate differentially expressed genes (DEGs) using the DESeq2R package with default parameters [15]. Then, the resulting *p*-values were adjusted across all candidate DEGs by applying the false discovery rate (FDR) to control false discovery rates. Finally, DEGs were defined with the absolute value of fold-change ≥ 2 and false discovery rate (FDR) ≤ 0.05 [16]. To ensure the accuracy of transcriptomic analysis, all sequence alignment, assembly, and annotation processes were based on the genome-wide data of *Chilo suppressalis* (taxid: 168631).

### 2.5. Real-Time Quantitative PCR

To verify the reliability of the RNA-seq data, the transcript levels of 8 representative DEGs were randomly chosen for real-time quantitative PCR (RT-qPCR) analysis. The primers (Table S1) for DEGs were designed using Oligo 7 software, and specifications were confirmed by NCBI Primer-BLAST (https://www.ncbi.nlm.nih.gov/tools/primer-blast/). Real-time PCR reactions were carried out as follows: 10 μL STBR Premix Ex Taq (2×) (Bio-Rad, Hercules, CA, USA); 2 μL cDNA templates; 6 μL ddH_2_O; 1 μL forward primer (10 μM); and 1 μL reverse primer (10 μM). Three biological replicates of each treatment and each gene were examined by RT-qPCR in quadruplicate. Relative changes in target gene expression were quantified using the 2^−ΔΔCt^ method, and tubulin served as a reference gene [17].

### 2.6. ER Activity and Cytosolic Ca^2+^ Concentration Assay

*Chilo suppressalis* larvae parasitized for 3 days were injected with the HSF1 inhibitor KRIBB11 (LC50 of 1.2 μM) configured with dimethyl sulfoxide. After 24 h of injection, host hemolymph was collected into tubes containing 200 μL of PBS buffer (containing 0.025% phenylthiourea) and centrifuged at 1000× *g* for 5 min at 4 °C to collect hemolymph cells. After centrifugation, the cells were resuspended and stained with 5 μL ER-Tracker Red working solution, 100 μL Fluo-4 AM, and 5 μL Hoechst staining solution for 30 min in the dark at 37 °C (Biyuntian, Suzhou, China). Finally, the smears were observed and photographed under a fluorescent microscope (Nikon, Ts2R, Tokyo, Japan). Images were analyzed using ImageJ Software (version 1.52: Bethesda, MD, USA). The cells collected from unparasitized and noninjected HSF1 inhibitor larvae were used as controls.

### 2.7. Statistical Analyses

Data were analyzed using one-way analysis of variance (ANOVA) followed by Duncan’s multiple comparison test. SPSS v. 20.0 software (IBM, Armonk, NY, USA) was used for statistical analyses, and results are presented as means ± SE (standard error). Data were considered significant at *p* < 0.05.

## 3. Results

### 3.1. SCP Values of Host Larvae After Parasitism

**Figure 1:**
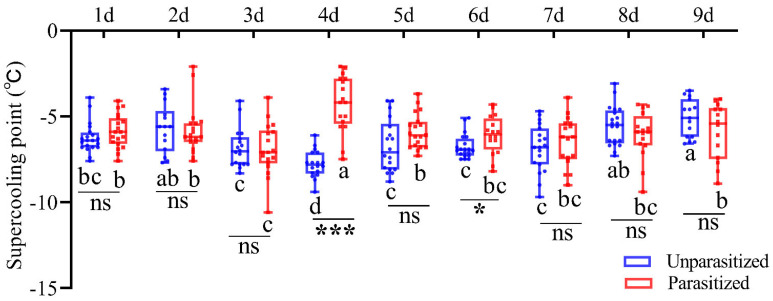
Determination of the supercooling points of *Chilo suppressalis* either unparasitized (blue boxes) or parasitized (red boxes) by *Cotesia chilonis*. Means marked with the same letter are not significantly different (Duncan’s multiple comparison test: *p* > 0.05). Asterisks indicate significant differences between temperatures (*t*-tests: * 0.01 < *p* < 0.05, *** *p* < 0.001), and ns (*p* ≥ 0.05).

SCPs of *C. suppressalis* larvae were measured after being parasitized for different periods of time (Figure 1). The results showed that the SCP of host larvae reached its lowest value after 3 days of parasitism and rebounded significantly on the fourth day, after which there was no significant difference with the duration of parasitism (*F*_8,146_ = 4.902, *p* < 0.001). Meanwhile, the SCP of unparasitized larvae changed significantly with their own developmental process (*F*_8,153_ = 8.774, *p* < 0.001). Interestingly, the SCP of *C. suppressalis* larvae was significantly higher after 4 and 6 days of parasitism compared to unparasitized larvae (4 days: *t* = 8.028, *p* < 0.001; 6 days: *t* = 2.192, *p* = 0.035).

### 3.2. mRNA Sequencing of the Larval Transcriptome

RNA-seq sequencing analysis was performed on larvae parasitized for 3 and 4 days (P3d and P4d) and compared with unparasitized larvae as control (CK). The clean reads of each sample were individually sequence-aligned with the reference genome of *C. suppressalis* (taxid: 168631) [18]. High-quality clean reads were obtained from raw reads by removing low-quality reads, with clean reads comprising more than 96% of raw reads in each sample. Then, clean reads from all groups were mapped to the reference genome, and the total mapping rate reached 61.93–77.38%. After quality control, the GC percentages and Q20 were 42–46% and 96.38–97.75%, respectively (Table 1).

### 3.3. Differential Gene Expression

**Figure 2:**
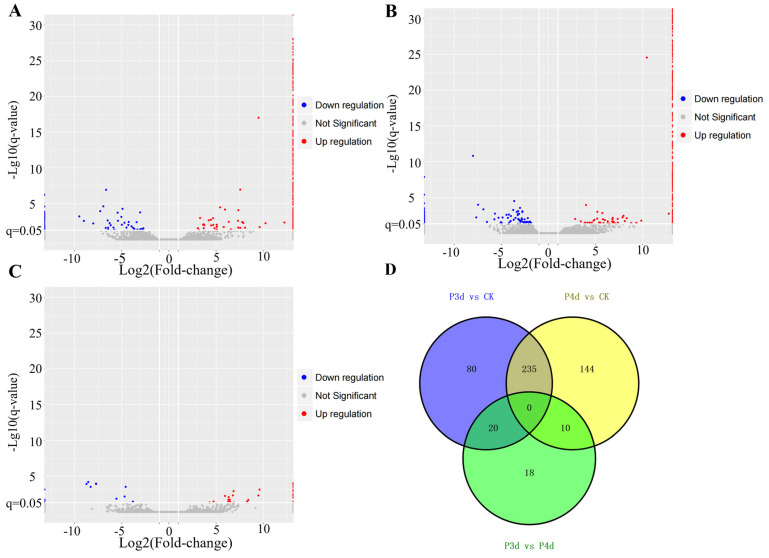
Differentially expressed genes (DEGs) in *Chilo suppressalis* transcriptomes. Volcano plots of (**A**) P3d vs. CK, (**B**) P4d vs. CK, (**C**) P4d vs. P3d, and Venn diagram of (**D**).

Pairwise comparison of transcriptomes between parasitism treatment groups and control (P3d vs. CK, P4d vs. CK, and P4d vs. P3d) indicated that 335 (264 up- and 71 down-regulated), 389 (290 up- and 99 down-regulated), and 48 (33 up- and 15 down-regulated) unigenes, respectively, were DEGs (Figure 2A–C). Regarding the cluster analysis of DEGs among the different treatment groups, there were no common DEGs among the three treatment groups, and two-by-two cluster analysis revealed 10, 20, and 235 common DEGs, respectively (Figure 2D).

**Figure 3:**
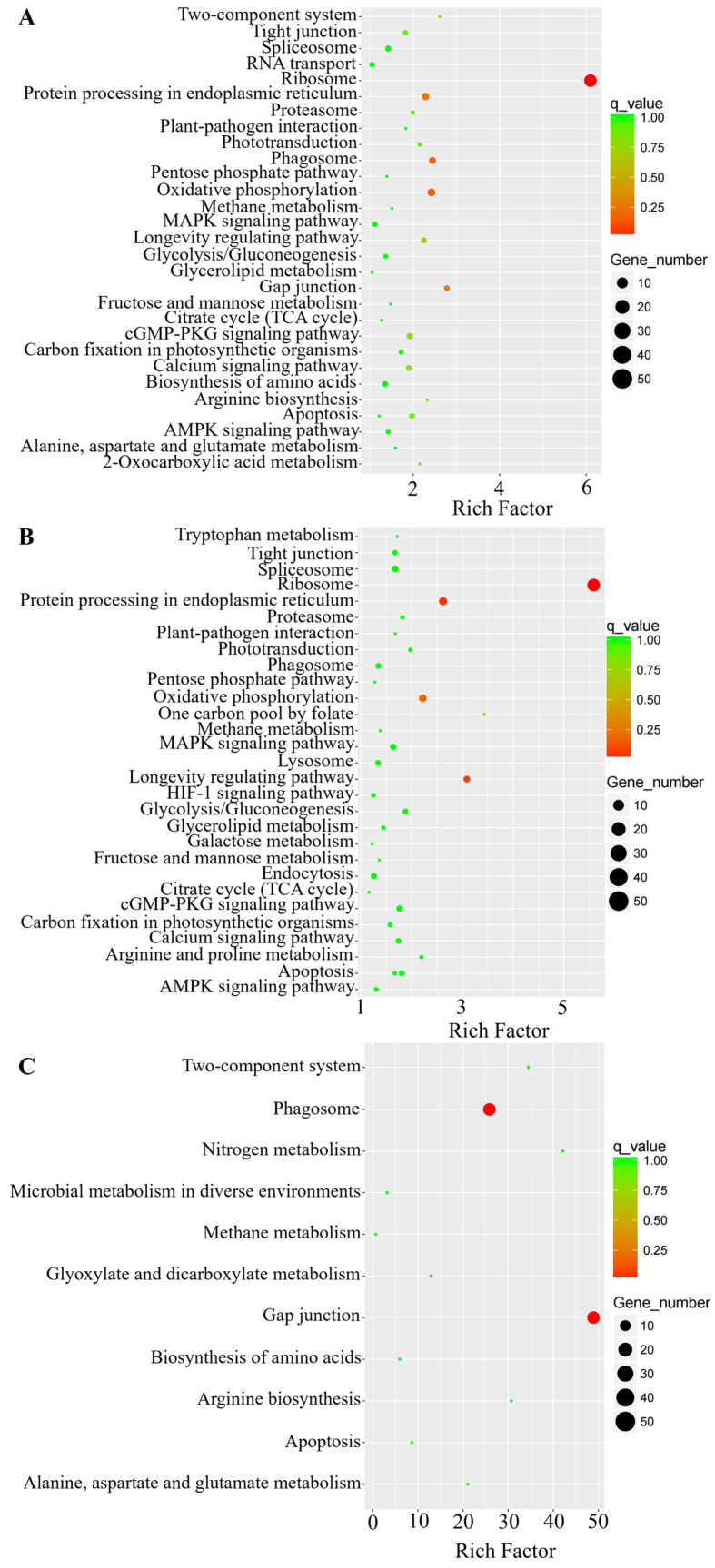
The 30 most enriched KEGG pathways among DEGs. The *x*-axis represents the level of Rich factor, and *y*-axis represents the type of pathway. Dot size represents the number of DEGs, and colors indicate corrected *p*-values. (**A**) P3d vs. CK, (**B**) P4d vs. CK, and (**C**) P4d vs. P3d.

The molecular functions involved in DEGs were further analyzed using the KEGG database, and the 30 most significantly enriched KEGG pathways are shown in Figure 3. The results of the enrichment analysis indicated that DEGs were mainly related to ribosome, protein processing in endoplasmic reticulum, and oxidative phosphorylation functions during parasitism stress (Figure 3A,B). On the other hand, with prolonged parasitism, DEGs were mainly related to phagosome and gap junction functions (Figure 3C).

### 3.4. Expression Patterns of Temperature Tolerance Associated Genes

Twenty-four DEGs related to temperature tolerance were identified and annotated, of which 21 and 3 genes were up- and down-regulated, respectively (Table 2). Analysis revealed that numerous heat shock proteins (HSPs) and calcium signaling-related genes linked to host temperature tolerance were significantly up-regulated under parasitism stress. Conversely, two small heat stress proteins (*sHsp20* and *sHsp21.3*) and a cuticle protein gene were significantly down-regulated. In addition, DEGs associated with temperature tolerance were involved in endoplasmic reticulum function and stabilization of the intracellular environment.

### 3.5. Transcriptional Profiles of Selected Genes by RT-qPCR

**Figure 4:**
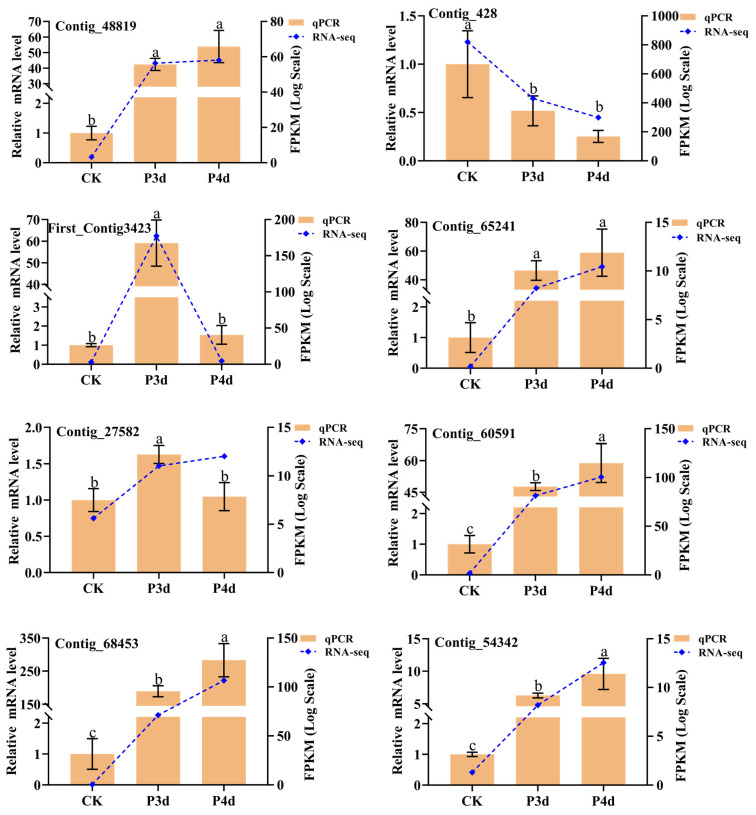
Validation of gene expression by RT-qPCR of selected genes. Different lowercase letters indicate significant differences among treatments (*p* < 0.05). Orange bars indicate the relative expression level (*y*-axis on left), and the blue lines represent the FPKM values (*y*-axis on right).

RT-qPCR was performed to validate eight DEGs obtained from RNA-seq data (Figure 4). The results showed that all genes except *sHsp20* gene (Contig_27582) exhibited the same expression trend as RNA-seq data. On the fourth day of parasitism, the *sHsp21.3* gene (Contig_428) was significantly suppressed in the host. The cuticle protein gene (First_Contig3423) showed a trend of increasing and then decreasing. On the other hand, five genes associated with ER function (Contig_68453, Contig_54342, and Contig_48819) and stabilization of the intracellular environment (Contig_65241 and Contig_60591) showed significant up-regulation with prolonged parasitism compared with CK treatment.

### 3.6. Detection of ER Activity and Cytosolic Ca^2+^ Concentration

**Figure 5:**
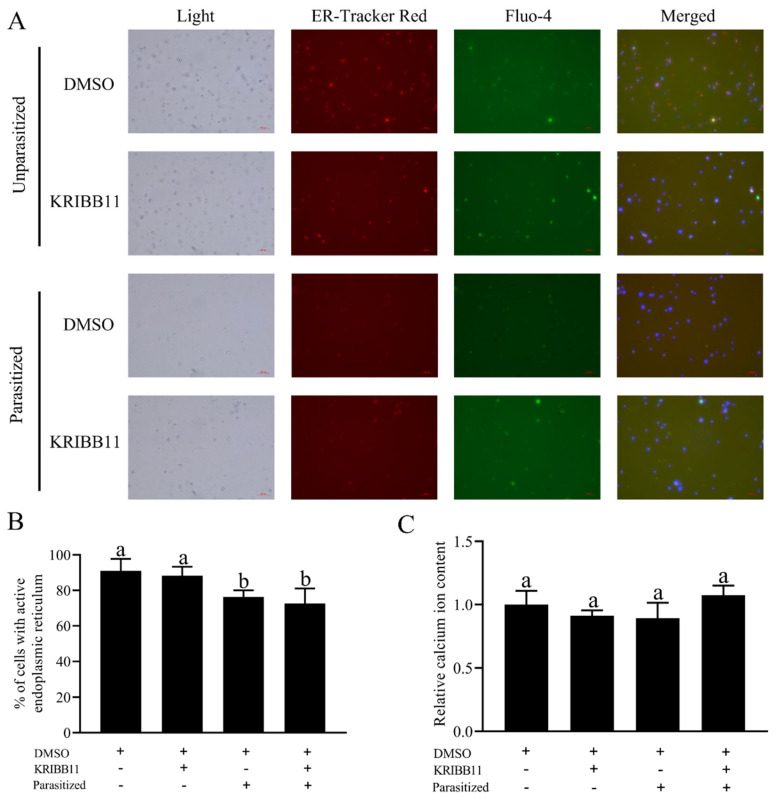
Effect of parasitic stress on endoplasmic reticulum activity and Ca^2+^ concentration in hemolymph cells of *Chilo suppressalis*. (**A**) Representative fluorescence images from each group. (**B**) Percentage of cells with active endoplasmic reticulum. (**C**) Relative Ca^2+^ concentration. Different lowercase letters indicate significant differences among treatments (*p* < 0.05).

The ER activity and cytosolic Ca^2+^ concentration were measured in hemolymph cells of parasitized *C. suppressalis* larvae after injection of HSF1 inhibitor (Figure 5A). The results showed that parasitism stress had a significant inhibitory effect on ER activity in hemolymph cells of *C. suppressalis* larvae, but no significant effect on the cytosolic Ca^2+^ concentration (Figure 5B). On the other hand, injection of HSF1 inhibitor had no significant effect on cytosolic Ca^2+^ concentration in hemolymph cells of *C. suppressalis* larvae (Figure 5C). The above results indicate that parasitism stress only had a significant inhibitory effect on ER activity in host hemolymph cells

## 4. Discussion

As poikilotherms, insects have evolved physiological and behavioral strategies to survive winter; they can either tolerate freezing of extracellular fluid (freeze tolerance) or can reduce the freezing point of extracellular fluid to avoid freezing (freeze avoidance) [19]. An important consideration in insect cold tolerance is the supercooling point (SCP), which is affected by a range of variables, including body size, developmental stage, and weight, as well as rearing conditions, external temperature, and photoperiod [20,21]. In this study, the potential effects of parasitism stress on host cold tolerance were investigated by determining changes in the SCP of parasitized *C. suppressalis* larvae. The results indicated that the SCP of host larvae was lowest after 3 days of parasitism, rebounded significantly on the 4th day, and then remained stable (Figure 1). Furthermore, the SCP is implicated in physiological processes such as the accumulation of cryoprotectants, the elimination of ice nucleating substances, and the synthesis of HSPs in insects [22,23]. This is consistent with our transcriptome data, which revealed an alteration in *C. suppressalis* larvae SCP accompanied by changes in gene expression (Table 2). Research has demonstrated that the lipid content of *Drosophila melanogaster* varies with the duration of exposure to a temperature environment [24]. In *Harmonia axyridis*, a notable elevation in glycogen content and soluble trehalase activity contributes to enhanced cold tolerance and facilitates overwintering [25].

Transcriptome analysis of *C. suppressalis* showed that numerous HSPs were induced to be expressed under parasitism stress in *C. chilonis* (Table 2). Numerous studies have found that HSPs are synthesized by cells or organisms under the action of heat shock and other stress factors, and are mainly divided into four major families, such as *HSP90*, *HSP70*, *HSP60*, and small heat shock proteins (sHSPs), depending on protein size and relatedness [26,27]. It has been shown that sHSPs are able to protect cells against protein aggregation and proteotoxic stress by disrupting the association with trapped misfolded proteins and delivering misfolded proteins to autophagosomes in the involvement of HSP70 [28]. In addition, the interactions between sHSPs and other chaperones of the ATP-dependent *Hsp70* and *Hsp90* lead to protein homeostasis, ensuring efficient substrate solubilization and refolding of damaged proteins [29,30]. In this study, we found that the expression of *HSP70* and *HSP90* was significantly induced in parasitized *C. suppressalis*, whereas the diametrically opposite situation was observed in sHSPs. The synchrony of transcript levels of various HSP genes may constitute a significant factor contributing to the loss of protein function and the altered cold tolerance observed in *C. suppressalis*.

This study demonstrated that DEGs were primarily enriched in the “phagosome” and “gap junction” of the KEGG pathway (Figure 3). Phagosomes are essential organelles that play a pivotal role in the immune response and the degradation of intracellular substances, with their functionality being contingent upon interactions with other organelles [31]. Parasitoids demonstrate a diverse array of parasitism mechanisms, which encompass the injection of various parasitic factors that suppress the host’s immune response and induce apoptosis [32]. Research has indicated that *Microplitis bicoloratus* specifically inhibits mitochondrial activity and triggers apoptosis in the hemolymph cells of the host [33]. This indicates that there are significant interactions between different organelles and even cells during the immune response induced by parasitism stress in *C. suppressalis*, potentially implicating the role of gap junction functions.

The oxidative property and elevated concentration of Ca^2+^ that characterize the internal environment of the endoplasmic reticulum (ER) are essential for protein synthesis, protein post-translational modification, and trafficking [34]. When the internal environment of the ER becomes imbalanced due to the accumulation of large amounts of misfolded proteins, it is referred to as endoplasmic reticulum stress (ER stress) [35]. Meanwhile, the chaperone proteins, especially the HSPs, are involved in the refolding of misfolded proteins and inhibiting the progress of protein synthesis to reduce the level of ER stress [36,37]. However, the transcription of HSPs is in turn regulated by heat shock factor (HSF), and once the transcriptional regulation is disrupted, the ER function and cytosolic Ca^2+^ concentration may be altered. In this study, we observed that a large number of genes associated with Ca^2+^ signaling were up-regulated (e.g., Calmodulin, Calcium-transporting ATPase, and Calcium-transporting ATPase sarcoplasmic) in parallel with the activation of HSPs (Table 2), suggesting a possible functional link between the two. The functional verification results show that parasitism stress had a significant inhibitory effect on ER activity (Figure 5), but the link between HSPs and ER function needs to be further investigated.

Parasitoid larvae primarily consume host hemolymph during the initial stages of their embryonic development, and they regulate the synthesis of low molecular sugars in hemolymph to promote their own growth and alter the host’s cold tolerance [38,39]. In the advanced stages of development, parasitoid larvae feed directly on the host’s fat body, which serves as a crucial energy store and as a metabolic organ, consequently diminishing the host’s tolerance to low temperatures [40,41]. The above results indicate that the changes in cold tolerance of *C. suppressalis* caused by *C. chilonis* involve a complex regulatory network.

## 5. Conclusions

This study reveals that parasitism by *Cotesia chilonis* modulates cold tolerance in *Chilo suppressalis* larvae, which is associated with transcriptomic changes, particularly in ribosome biogenesis and ER function. Functional assays showed parasitism inhibits ER activity, but not via HSPs or calcium signaling pathways. These findings highlight complex molecular mechanisms underlying host responses to parasitoid manipulation, especially concerning cold tolerance.

## Figures and Tables

**Table 1:** Quality control results of sequence data.

Sample ID	Raw Reads	Clean Reads	Mapped Reads	Clean/Raw (%)	Mapping Ratio (%)	GC Content (%)	Q20 Percentage (%)
CK-1	39,298,080	37,947,750	29,348,812	96.56	77.38%	45	96.5
CK-2	40,288,974	38,875,529	24,922,402	96.49	64.15%	43	96.38
CK-3	38,032,526	36,554,305	27,541,204	96.11	75.39%	46	96.39
P3d-1	40,815,776	39,096,635	25,993,339	95.79	66.56%	44	97.47
P3d-2	40,568,982	39,148,395	29,423,481	96.50	75.24%	45	97.5
P3d-3	41,113,492	39,735,610	24,592,438	96.65	61.93%	42	97.61
P4d-1	35,184,070	34,032,418	21,634,570	96.73	63.61%	43	97.72
P4d-2	38,969,532	37,868,907	27,525,104	97.18	72.73%	44	97.72
P4d-3	36,991,348	36,000,191	22,920,868	97.32	63.70%	43	97.75

**Table 2:** Annotation information on temperature tolerance-associated genes in *Chilo suppressalis*.

Internal ID	P3d vs. CK		P4d vs. CK		P4d vs. P3d		Annotation	Best Blast Hit Species
	log_2_FC	*p* Value	log_2_FC	*p* Value	log_2_FC	*p* Value		
Up-regulated gene								
contig_67252	81.84	1.38 × 10^−16^	99.14	1.19 × 10^−15^	0.28	7.16 × 10^−1^	Heat shock protein 90	*Lacanobia wlatinum*
contig_53467	294.18	2.45 × 10^−26^	505.65	4.38 × 10^−33^	0.78	1.99 × 10^−1^	Heat shock protein 70	*Melitaea cinxia*
contig_62749	280.06	9.52 × 10^−28^	480.18	3.18 × 10^−32^	0.78	2.05 × 10^−1^	Heat shock protein 70	*Cataclysta lemnata*
contig_45562	103.13	1.56 × 10^−27^	175.89	1.24 × 10^−34^	0.77	2.07 × 10^−1^	Heat shock protein 70	*Globia sparganii*
contig_66597	2.32	7.42 × 10^−3^	15.02	2.51 × 10^−9^	2.70	1.83 × 10^−3^	Heat shock protein 70	*Globia sparganii*
contig_66711	31.82	8.47 × 10^−7^	68.10	2.91 × 10^−11^	1.10	2.82 × 10^−1^	Heat shock protein 70	*Cotesia rubecula*
contig_48668	1.95	5.40 × 10^−3^	10.02	3.16 × 10^−8^	2.36	9.82 × 10^−3^	Heat shock protein 70	*Galleria mellonella*
contig_53496	43.34	1.87 × 10^−17^	85.32	2.12 × 10^−25^	0.98	1.23 × 10^−1^	Hsc70-3 protein	*Galleria mellonella*
contig_60591	64.80	4.19 × 10^−16^	80.53	3.13 × 10^−18^	0.31	6.50 × 10^−1^	Activating transcription factor of chaperone	*Diatraea saccharalis*
contig_46567	64.61	3.46 × 10^−17^	132.34	9.84 × 10^−26^	1.03	7.89 × 10^−2^	Cathepsin L Protein	*Fopius arisanus*
contig_53686	18.69	1.17 × 10^−6^	29.05	2.42 × 10^−9^	0.64	4.22 × 10^−1^	Actin	*Scoparia ambigua*
contig_62212	25.37	7.96 × 10^−9^	27.03	2.48 × 10^−11^	0.09	8.76 × 10^−1^	Translocator protein	*Melipona quadrifasciata*
contig_44603	24.51	3.57 × 10^−11^	35.70	8.64 × 10^−14^	0.54	4.32 × 10^−1^	AlphaTub84B	*Drosophila yakuba*
contig_65241	5.57	8.07 × 10^−3^	10.44	7.57 × 10^−6^	0.91	4.45 × 10^−1^	Apoptosis-inducing factor 1	*Microplitis demolitor*
contig_71196	4.69	1.98 × 10^−3^	10.23	5.52 × 10^−6^	1.12	2.37 × 10^−1^	Sterile 20-like protein	*Microplitis demolitor*
contig_68453	51.24	2.28 × 10^−9^	119.92	1.59 × 10^−22^	1.23	1.37 × 10^−1^	Calreticulin	*Helicoverpa zea*
contig_48819	35.71	3.23 × 10^−9^	50.13	1.83 × 10^−15^	0.49	5.73 × 10^−1^	Calmodulin	*Cyphomyrmex costatus*
contig_69274	9.50	2.80 × 10^−5^	11.23	4.80 × 10^−6^	0.24	7.87 × 10^−1^	Calcium-transporting ATPase	*Helicoverpa zea*
contig_59131	4.97	8.38 × 10^−5^	8.01	2.31 × 10^−6^	0.69	4.64 × 10^−1^	Calcium-transporting ATPase	*Nasonia vitripennis*
contig_6088	−9.01	1.23 × 10^−5^	0.60	7.05 × 10^−1^	9.60	2.03 × 10^−7^	Alpha-tubulin	*Ostrinia furnacalis*
contig_54342	6.86	1.47 × 10^−6^	9.86	3.12 × 10^−8^	0.52	5.20 × 10^−1^	Calcium-transporting ATPase sarcoplasmic	*/*
Down-regulated gene								
contig_428	−1.06	6.46 × 10^−2^	−1.99	1.71 × 10^−4^	−0.92	1.38 × 10^−1^	Heat shock protein 21.3	*Chilo suppressalis*
contig_27582	4.86	5.74 × 10^−2^	4.60	2.51 × 10^−4^	−0.25	8.86 × 10^−1^	Heat shock protein 20	*Bicyclus anynana*
First_Contig3423	4.68	4.86 × 10^−6^	−0.02	9.54 × 10^−1^	−4.70	3.60 × 10^−6^	Cuticle protein	*Amyelois transitella*

## Data Availability

The original contributions presented in this study are included in the article/Supplementary Material. Further inquiries can be directed to the corresponding authors.

## Supplementary Materials

The following supporting information can be downloaded at https://www.mdpi.com/article/10.3390/insects16090907/s1, Table S1. Primers used in this study.
