# Supplementary material for: Transcriptomic Analysis of Chilo suppressalis (Walker) (Lepidoptera: Pyralidae) Reveals Cold Tolerance Mechanisms Under Parasitism Stress
Source: Insects. 2025 Sep 1;16(9):907. doi: 10.3390/insects16090907 (PMC12470921; doi:10.3390/insects16090907)
Supplement: Supplementary file 1 [file insects-16-00907-s001.zip › insects-3746366-supplementary.pdf]

## Supplementary Materials

**Table S1.** Primers used in this study.

| Gene | | Primer sequences (5′→3′) |
|---|---|---|
| *contig_60591* | Forward | CGTGCATAGGGCTTATTGCGAGT |
| | Reverse | CCAGCAGTAGCAAGGCTAGCAGT |
| *contig_65241* | Forward | ACCCGCCACCGATAATGACG |
| | Reverse | CGACCCCCAAGATGCTAGACG |
| *contig_68453* | Forward | GCCACCCAAGAAAATCAAGGACCC |
| | Reverse | GGATAGTCTCAGGCTTGTCCCAGT |
| *contig_48819* | Forward | CTGTATCTTTCATTTTACGCGCCAT |
| | Reverse | ATCCAACGGAAGCTGAATTACAGGA |
| *contig_54342* | Forward | CGCCCTCGGTTTCAATCCACCC |
| | Reverse | AGCCACCAATAGCGAGGTAACGG |
| *contig_428* | Forward | TGAGAACCATCAATTACACTTGAACC |
| | Reverse | CTTGCGTTATACTGAGTCGAG |
| *contig_27582* | Forward | GCCAAATGAAACCAGAGGGA |
| | Reverse | TCGCTGAGATTTACGATGTTGTTT |
| *Contig3423* | Forward | GGCGAGGATAATCATTGACTGC |
| | Reverse | GGCGAAAATTTCTAGGCGAGTACTGG |
| *Tubulin (TUB)* | Forward | GAGGGCATGGACGAGATGGA |
| | Reverse | ACGACGGTACGAGTATGACGGG |
